# Editorial: Recent advances in theranostics: endocrine tumours and beyond

**DOI:** 10.3389/fmed.2026.1975494

**Published:** 2026-09-02

**Authors:** Alberto Nieri, Alessio Imperiale, Luca Urso

**Affiliations:** 1 Nuclear Medicine Unit, Oncological Department, Azienda Ospedaliero Universitaria Senese, Siena, Italy; 2 Nuclear Medicine, Nouvel Hôpital Civil, University Hospitals of Strasbourg, University of Strasbourg, Strasbourg, France; 3 Department of Translational Medicine, University of Ferrara, Ferrara, Italy; 4 Nuclear Medicine Unit, Onco-Hematology Department, University Hospital of Ferrara, Ferrara, Italy

**Keywords:** neuroendocrine tumor, prostate cancer, radioiodine (131I) treatment, radionuclide, skin cancer, theranostic

In recent decades, radionuclide therapy has undergone substantial development, proving effective in the treatment of various oncological conditions. Available therapies range from established treatments, such as radioiodine therapy for differentiated thyroid cancer (1), to more recently developed approaches for neuroendocrine tumours (NETs) (2, 3), prostate cancer (4), and cutaneous malignancies (5). In this context, theranostics—the ability to use the same molecular carrier for both diagnostic characterization and targeted therapy—represents an increasingly relevant approach in nuclear medicine. Recent innovations focus on refining patient selection and tailoring treatment to individual disease biology, with the aim of improving clinical outcomes by integrating biological, imaging, and clinical parameters that were previously inaccessible. From this perspective, theranostics represents one of the most advanced expressions of precision oncology.

This Research Topic, “Recent Advances in Theranostics: Endocrine Tumours and Beyond”, includes five contributions that, taken together, highlight potential new directions in this rapidly evolving field. Rather than representing a single disease or therapeutic strategy, these studies illustrate different aspects of contemporary theranostics, ranging from the identification of biomarkers predictive of treatment response to the optimization of functional imaging, the development of alternative radiopharmaceutical approaches, and the refinement of radionuclide treatment strategies in advanced disease.

With regard to radioiodine therapy (RAI) for differentiated thyroid cancer, Qin et al. investigated whether the assessment of circulating biomarkers, specifically serum cytokines and serum thyroglobulin (sTg), could provide predictive information regarding the response to initial RAI in patients with intermediate- and high-risk disease. In a retrospective cohort of 429 patients, divided into a training cohort of 300 patients and an external validation cohort of 129 patients, sTg and interferon-alpha (IFN-α) were identified as independent predictors of treatment response. Based on these variables, the authors developed and externally validated a nomogram for predicting response to initial RAI. The predictive performance was good, with a C-index of 0.750 in the training cohort and 0.693 in the validation cohort, and the study highlights the potential value of integrating biochemical and immunological parameters into conventional risk assessment. This approach may contribute to earlier identification of patients who require closer follow-up or additional therapeutic considerations.

The importance of selecting the appropriate molecular imaging strategy is further illustrated by the study of Tepede et al., which evaluated the efficacy of [^18^F]F-DOPA PET/CT (DOPA-PET) in identifying NETs in patients with multiple endocrine neoplasia type 1 (MEN1) compared to conventional imaging and [^68^Ga]Ga-DOTATATE PET/CT (DOTA-PET). In a prospective cohort of 16 genetically confirmed MEN1 patients, 134 lesions were identified using computed tomography (CT), magnetic resonance imaging (MRI) and DOTA-PET, whereas DOPA-PET detected 23 lesions, corresponding to 17% of the total. Overall, the authors concluded that DOPA-PET has low efficacy for the detection of MEN1-related NETs. Nevertheless, authors point out that, in selected clinical situations, including serotonin-secreting lesions, equivocal SSTR imaging, or specific functional tumours, DOPA-PET may provide complementary information. Thus, these findings reinforce the concept that imaging should increasingly be adapted to the biological characteristics of individual tumours rather than relying on a single imaging strategy, within a patient tailored oncological approach.

Considering the growing development of theranostic aspects in the field of prostate cancer, Albano et al. performed a systematic review and meta-analysis evaluating the diagnostic role of [^99m^Tc]Tc-PSMA SPECT/CT. Twenty-three studies involving 1,840 patients were included, with 19 studies contributing to the meta-analysis. The pooled detection rate (DR) was 79.7% in the staging setting and 75.4% in patients with biochemical recurrence. Importantly, PSA levels were frequently associated with the DR. Although significant heterogeneity was observed among the included studies, these results support the potential role of SPECT/CT as an alternative diagnostic approach, particularly in clinical contexts worldwide where PET/CT is not readily available (6). This aspect is particularly relevant because the future development of theranostics will depend not only on biological effectiveness but also on the possibility of making advanced molecular imaging and targeted treatments accessible across different healthcare environments (7).

The therapeutic counterpart of PSMA-targeted imaging is discussed in the narrative review by Gierulska et al., which summarizes current evidence on [^177^Lu]Lu-PSMA-targeted radioligand therapy (RLT), emerging [^225^Ac]Ac-PSMA strategies, and therapeutic sequencing in advanced prostate cancer. The review highlights the established role of [^177^Lu]Lu-PSMA in selected patients with metastatic castration-resistant prostate cancer (mCRPC), as demonstrated by pivotal trials including VISION, TheraP, and PSMAfore (4, 8, 9). In particular, the PSMAfore trial supports the potential benefit of introducing [^177^Lu]Lu-PSMA RLT earlier in the disease course, before taxane-based chemotherapy, in selected taxane-naïve patients. At the same time, emerging alpha-emitting approaches such as [^225^Ac]Ac-PSMA may offer additional therapeutic opportunities, although definitive phase III evidence is still lacking. These developments underline that theranostics is progressively moving towards the optimization of treatment timing, radionuclide selection, toxicity management, and therapeutic sequencing.

Finally, Sadeghpour et al. broadened the application of targeted radionuclide therapy to cutaneous malignancies by systematically reviewing the available evidence on rhenium-assisted therapy for skin lesions. Their review and meta-analysis included 10 studies, encompassing a total of 433 patients and over 618 lesions. Among the seven studies included in the meta-analysis, rhenium-assisted therapy achieved a pooled complete response rate of 88.67% and an overall response rate of 92.9%. Although these findings are encouraging, the authors appropriately emphasize the need for further studies and standardized treatment protocols. This contribution is particularly relevant in demonstrating how radionuclide-based approaches may extend beyond the more established fields of endocrine and prostate malignancies and potentially provide minimally invasive therapeutic options for selected patients with cutaneous tumours.

Taken together, the five contributions included in this Research Topic reflect several complementary aspects of the current evolution of theranostics. The studies by Qin et al. and Tepede et al. emphasize the importance of biological characterization and appropriate molecular imaging in endocrine tumours, while the contributions addressing prostate cancer demonstrate how target-based imaging can be integrated with therapeutic radionuclide delivery and increasingly sophisticated treatment sequencing. The systematic review of rhenium-assisted therapy further illustrates the expanding range of emerging potential applications of targeted radionuclide treatment.

A central challenge for the next phase of theranostics will be to move beyond a binary assessment of target expression. Molecular imaging should increasingly be regarded as a dynamic biomarker, capable of revealing spatial and temporal heterogeneity and changes induced by disease progression or treatment. Longitudinal imaging could therefore guide patient selection, treatment adaptation, and the identification of patients who may benefit from alternative radiopharmaceuticals or combined therapeutic strategies.

Future progress will also depend on integrating molecular imaging with circulating biomarkers, molecular profiling, conventional imaging, and patient-specific dosimetry (10). Such integration could support a transition from standardized treatment schedules towards more individualized decisions regarding administered activity, treatment intervals, number of cycles, radionuclide selection, and therapeutic sequencing. Advanced quantitative imaging and artificial intelligence may facilitate this process, provided that these approaches undergo robust multicentre validation and methodological harmonization.

Finally, innovation in theranostics must be accompanied by attention to clinical evidence, feasibility, and equitable access. Prospective comparative trials, standardized response criteria, long-term toxicity assessment, and health-economic analyses will be essential to determine the added value of emerging strategies (11). The maturation of theranostics will depend not only on new radiopharmaceuticals, but also on their integration into multidisciplinary, evidence-based, and widely implementable clinical pathways.
